# Potential Biomarkers for Post-Stroke Cognitive Impairment: A Systematic Review and Meta-Analysis

**DOI:** 10.3390/ijms23020602

**Published:** 2022-01-06

**Authors:** Ka Young Kim, Ki Young Shin, Keun-A Chang

**Affiliations:** 1 Department of Nursing, College of Nursing, Gachon University, Incheon 21936, Korea; kykim@gachon.ac.kr; 2 Neuroscience Research Institute, Gachon University, Incheon 21565, Korea; 3 Bio-MAX Institute, Seoul National University, Seoul 08826, Korea; 4 Department of Pharmacology, College of Medicine, Gachon University, Incheon 21936, Korea; 5 Neuroscience of Health Sciences and Technology, Gachon Advanced Institute for Health Sciences and Technology, Gachon University, Incheon 21936, Korea

**Keywords:** stroke, dementia, cognitive impairment, post-stroke cognitive impairment, blood biomarker

## Abstract

Stroke is a primary debilitating disease in adults, occurring in 15 million individuals each year and causing high mortality and disability rates. The latest estimate revealed that stroke is currently the second leading cause of death worldwide. Post-stroke cognitive impairment (PSCI), one of the major complications after stroke, is frequently underdiagnosed. However, stroke has been reported to increase the risk of cognitive impairment by at least five to eight times. In recent decades, peripheral blood molecular biomarkers for stroke have emerged as diagnostic, prognostic, and therapeutic targets. In this study, we aimed to evaluate some blood-derived proteins for stroke, especially related to brain damage and cognitive impairments, by conducting a systematic review and meta-analysis and discussing the possibility of these proteins as biomarkers for PSCI. Articles published before 26 July 2021 were searched in PubMed, Embase, the Web of Science, and the Cochrane Library to identify all relevant studies reporting blood biomarkers in patients with stroke. Among 1820 articles, 40 were finally identified for this study. We meta-analyzed eight peripheral biomarker candidates: homocysteine (Hcy), high-density lipoprotein cholesterol (HDL-C), C-reactive protein (CRP), low-density lipoprotein cholesterol (LDL-C), total cholesterol (TC), triglyceride (TG), uric acid, and glycated hemoglobin (HbA1c). The Hcy, CRP, TC, and LDL-C levels were significantly higher in patients with PSCI than in the non-PSCI group; however, the HDL-C, TG, uric acid, and HbA1c levels were not different between the two groups. Based on our findings, we suggest the Hcy, CRP, TC, and LDL-C as possible biomarkers in patients with post-stroke cognitive impairment. Thus, certain blood proteins could be suggested as effective biomarkers for PSCI.

## 1. Introduction

Stroke occurs in 15 million individuals each year, causing high mortality and disability rates. The latest estimate revealed that stroke is the second leading cause of death worldwide [1,2]. Most strokes are ischemic, owing to the presence of a reduced blood flow, generally resulting from arterial occlusion. The remaining 10–40% of stroke presentations are hemorrhagic, depending on regional epidemiology and resulting from the rupture of the cerebral arteries [3,4]. Structural damage to the brain in patients with stroke occurs because of both ischemia and hemorrhage [5,6,7]. As a result, even minor stroke affects daily functions, executive functions, and cognition, consequently affecting patients’ activity performance, quality of life, and ability to return to work [8,9].

In particular, the cognitive domains involved in the development of dementia after stroke may vary depending on the stroke characteristics, such as stroke type, volume, number, location, and severity [10,11]. (1) Important critical locations include the dominant hemisphere and lesions affecting the prefrontal–subcortical circuit that mediates executive dysfunction [12,13]. (2) Frontal lobe functions comprising processing speed, reaction time, working memory, and executive task measures are most commonly affected [14]. (3) A single large cortico-subcortical brain ischemic lesion may present with acute cognitive deterioration if located in an area that is functionally critical for cognition [15]. (4) Strategic infarction dementia may be caused by damage to the components of the Papez (hippocampal memory loop) [16] or Yokovlev circuits [11].

Generally, stroke diagnosis depends crucially on neuroimaging; computed tomography remains an essential component of stroke management, although it is not always available [17]. Over the last decades, molecular biomarkers for stroke have gained the attention of clinicians around the world owing to their broad application in facilitating diagnosis, characterizing clinical size and severity, estimating long-term prognoses, and selecting an appropriate treatment option [18,19]. The National Institutes of Health Biomarkers Definitions Working Group proposed a new definition of biomarkers in 2001: “a characteristic that is objectively measured and evaluated as an indicator of normal biological processes, pathogenic processes, or pharmacologic responses to a therapeutic intervention” [20,21]. Thus, biomarkers are beneficial for patients, caregivers, and clinicians for: (a) planning subsequent clinical pathways and goal setting; and (b) identifying whom and when to target and, in some instances, at which level to use, along with interventions for promoting stroke recovery [22]. The application of these biomarkers can improve risk stratification and therefore guide the implementation of tailored treatment modalities [23].

Unlike general stroke, post-stroke cognitive impairment (PSCI), one of the major complications after stroke, is frequently underdiagnosed, as it may be overlooked in the presence of other distressing signs (e.g., motor or visual symptoms). Consequently, the cognitive impact of acute stroke is often underestimated [24,25]. However, stroke has been reported to increase the risk of cognitive impairment by at least five to eight times [26]. To diagnose PSCI, in fact, neuroimaging features of computed tomography (CT) or magnetic resonance imaging (MRI) such as functional MRI (fMRI) and diffusion tensor imaging (DTI) have been used [27,28] and cognitive assessments such as the Information Questionnaire on Cognitive Decline in the Elderly (IQCODE), the Mini-Mental State Examination (MMSE), and the National Institute of Neurological Disease and Stroke (NINDS) battery have been applied [29].

Therefore, we aimed to evaluate some blood-derived proteins, especially those related to brain damage and cognitive impairments, by conducting a systematic review and meta-analysis and discussing the possibility of these proteins as biomarkers for PSCI.

## 2. Results

### 2.1. Characteristics of the Included Studies Reporting Potential Biomarkers for PSCI

**Figure 1:**
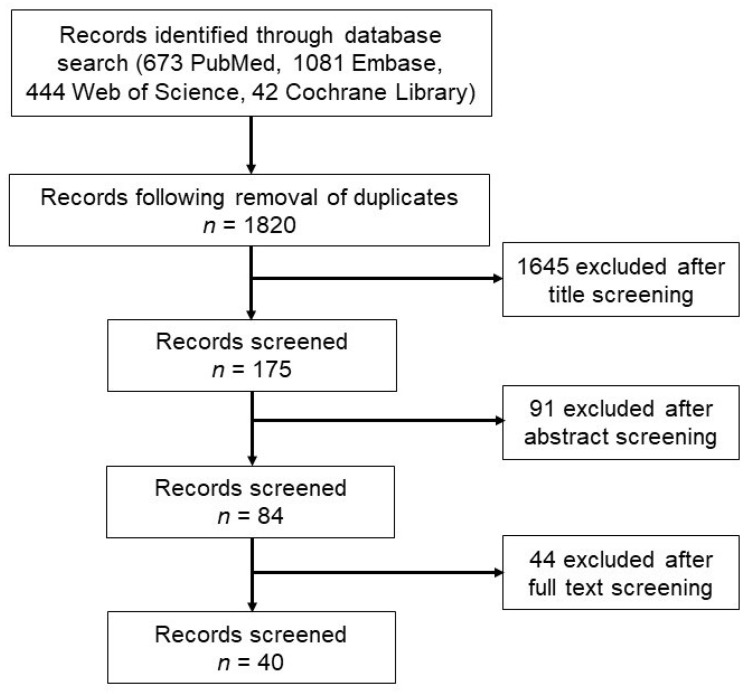
Flow diagram of the study selection.

We identified 2240 studies including 673 from PubMed, 1081 from Embase, 444 from the Web of Science, and 42 from the Cochrane Library (Figure 1). Duplicates were then excluded, yielding a total of 1820 studies. Thereafter, 84 studies were assessed for eligibility based on their title and abstract after excluding the following studies: (1) studies that did not investigate potential blood biomarkers for evaluating cognitive function after stroke, (2) studies that used cell or animal models, and (3) commentaries, letters, editorials, conference abstracts, or reviews. After a full-text review, 40 articles were finally included in this study.

Table 1 shows the selected studies that reported potential blood biomarkers for PSCI. The included studies were published between 2004 and 2021. The countries of patients were Lithuania, Argentina, Japan, Taiwan, the USA, France, China, Canada, Israel, Poland, the Russian Federation, Sweden, and Turkey. The types of groups used included post-stroke without dementia and post-stroke dementia groups, drug or related molecular concentration groups, stroke progression groups, and Mini-Mental State Examination (MMSE) level groups. The sample size of the case and control groups ranged from 8 to 1029, dividing male and female patients and presenting the mean age in each group. The outcome measurement tools used for evaluating cognitive impairment included the MMSE, Montreal Cognitive Assessment, and clinical dementia rating. Furthermore, the sample specimens used were the plasma or serum. Potential blood biomarkers were identified in the selected studies.

### 2.2. Classification of Potential Blood Biomarkers for PSCI

The potential blood biomarkers identified in the selected studies were categorized into blood and vascular functions, inflammatory and immune functions, metabolic function, neuronal function, kidney function, oxidative stress, hormones, and others (Table 2).

Of the identified potential biomarkers, the D-dimer, homocysteine (Hcy), endostatin, fibrinogen, vascular cell adhesion molecule 1 (VCAM-1), endogenous secretory receptor for advanced glycation end products (esRAGE), hs-CRP (high-sensitivity C-reactive protein) or CRP, indoleamine 2,3-dioxygenase, interleukin-10 (IL-10), interleukin-1 beta (IL-1β), interleukin-6 (IL-6), kynurenine, matrix metalloproteinase-9 (MMP-9), phospholipase A2, quinolinic acid, rheumatoid factor (RF), soluble receptor for advanced glycation end products (sRAGE), semicarbazide-sensitive amino oxidase, tissue inhibitor metalloproteinase-1 (TIMP-1), trimethylamine N-oxide (TMAO), tumor necrosis factor-alpha (TNF-α), kynurenine/tryptophan ratio, quinolinic acid/kynurenic acid ratio, fasting blood glucose (FBG), glycated hemoglobin (HbA1c), high-density lipoprotein cholesterol (HDL-C), low-density lipoprotein cholesterol (LDL-C), non-HDL-C, total cholesterol (TC), triglyceride (TG), β-secretase enzyme (BACE1), neprilysin, neurofilament light (NfL), cystatin C, uric acid, 8-hydroxydeoxyquanosine (8-OHdG), D-amino acid oxidase, malondialdehyde, N-terminal pro b-type natriuretic peptide (NT-proBNP), and cortisol levels increased among patients with PSCI.

Meanwhile, the butyrylcholinesterase (BChE), hs-CRP or CRP, sRAGE, betaine, TC, brain-derived neurotrophic factor (BDNF), amyloid beta 42 (Aβ42), Aβ42/amyloid beta (Aβ40), NfL, estimated glomerular filtration rat (eGFR), uric acid, 25-hydroxyvitamin D3 (25(OH)D), free thyroxin (FT4), triiodothyronine (T3), choline, formaldehyde, and NO^−2^ levels decreased.

The following extracted potential blood biomarkers did not change: direct bilirubin, fibrinogen, Hcy, indirect bilirubin, total bilirubin, tissue plasminogen activator, vitamin B12, vascular endothelial growth factor, von Willebrand factor, thrombin-antithrombin, anticardiolipin antibodies, IgG anticardiolipin antibodies units (aCL GPL), anti-phosphatidylserine antibodies, IgG antiphosphatidylserine antibodies units (aPS GPS), beta(2)-glycoprotein 1-dependent anticardiolipin antibodies (β2-GPI), complement component 3, CRP, kynurenic acid, lipoprotein-associated phospholipase A2 mass (Lp-PLA2 mass), tryptophan, interferon-gamma (IFN- γ), interleukin-1 receptor antagonist (IL-1 RA), IL-6, IL-8, IL-10, FBG, glucose, HbA1c, HDL-C, hepatocyte growth factor (HGF), LDL-C, TC, TG, insulin-like growth factor 1 (IGF-1), S100B protein, Aβ42, Aβ40, acetylcholinesterase (AChE), neprilysin, creatinine, uric acid, urea N, dehydroepiandrosterone sulphate (DS), free triiodothyroinine (FT3), NT-proBNP, thyroxin (T4), thyrotropin (TSH), Ca, folic acid, TMAO, retinoic acid, and cortisol/DS ratio.

### 2.3. Meta-Analysis Results of the Hcy, hs-CRP, Uric Acid, HbA1c, TC, TG, HDL-C, and LDL-C Levels

**Figure 2:**
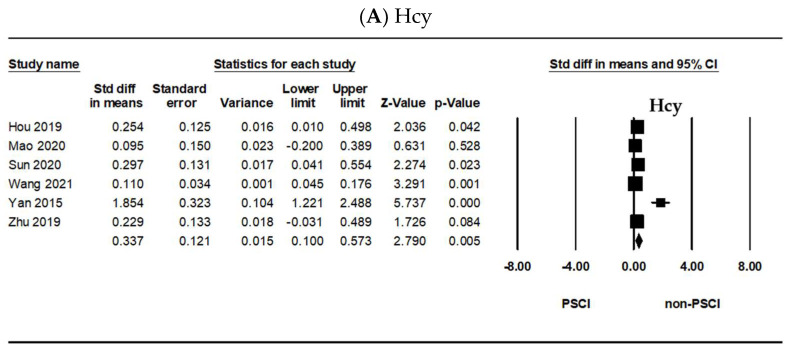

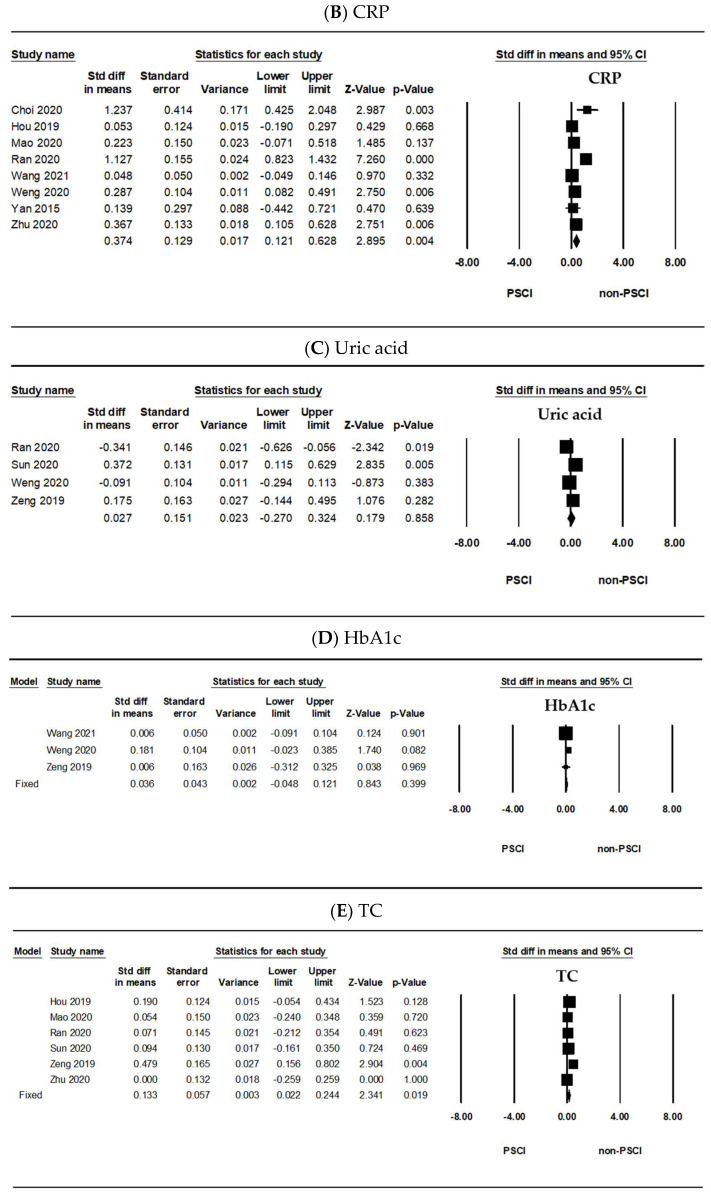

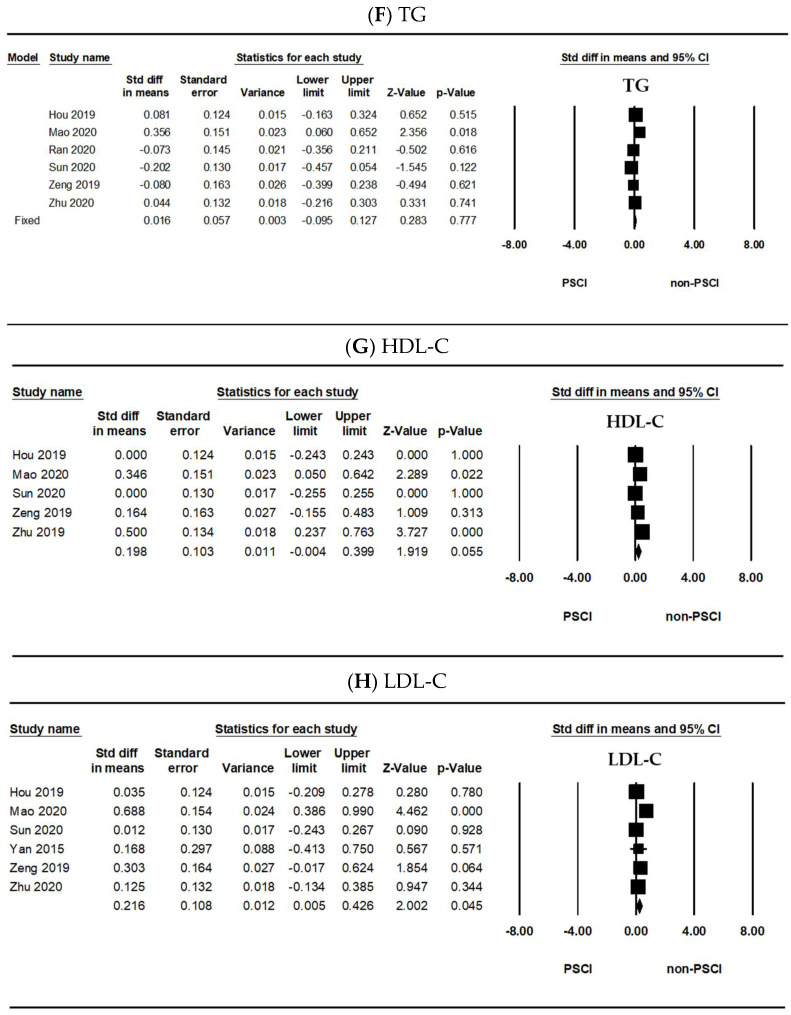
Forest plots of the (**A**) Hcy, (**B**) CRP, (**C**) uric acid, (**D**) HbA1c, (**E**) TC, (**F**) TG, (**G**) HDL-C, and (**H**) LDL-C levels. Hcy: homocysteine, CRP: C-reactive protein, HbA1c: glycated hemoglobin, TC: total cholesterol, TG: triglyceride, HDL-C: high-density lipoprotein cholesterol, LDL-C: low-density lipoprotein cholesterol, Std diff: standard difference, CI: confidence interval, PSCI: post-stroke cognitive impairment.

Figure 2 shows the meta-analysis results of the potential blood biomarkers between the PSCI and the non-PSCI group that were identified in five or more articles. As shown in Figure 2A, the Hcy level significantly differed between the PSCI and the non-PSCI groups (SMD = 0.337, 95% confidence interval (CI) = 0.100–0.573, *p* = 0.005). The CRP level also significantly differed between them (SMD = 0.374, 95% CI = 0.121–0.628, *p* = 0.004) (Figure 2B). Meanwhile, the uric acid (SMD = 0.027, 95% CI = −0.270–0.324, *p* = 0.858) (Figure 2C) and HbA1c levels (SMD = 0.036, 95% CI = −0.048–0.121, *p* = 0.399) (Figure 2D) did not significantly differ between the two groups. Furthermore, the TC level significantly differed between the PSCI and the non-PSCI groups (SMD = 0.133, 95% CI = 0.022–0.244, *p* = 0.019) (Figure 2E). The TG (SMD = 0.016, 95% CI = −0.095–0.127, *p* = 0.777) (Figure 2F) and HDL-C levels (SMD = 0.198, 95% CI = −0.004–0.399, *p* = 0.055) (Figure 2G) did not significantly differ between them. As shown in Figure 2H, the LDL-C level significantly differed between the two groups (SMD = 0.216, 95% CI = 0.005–0.426, *p* = 0.045).

As shown in Figure 2A (*I*^2^ = 84%, *p* < 0.001), 2B (*I*^2^ = 87%, *p* < 0.001), 2C (*I*^2^ = 81%, *p* = 0.002), 2G (*I*^2^ = 63%, *p* = 0.028), and 2H (*I*^2^ = 66%, p = 0.012), the heterogeneity was significant; thus, we used the random-effects model. The fixed-effects model was applied in Figure 2D (*I*^2^ = 14%, *p* = 0.311), 2E (*I*^2^ = 19%, *p* = 0.291), and 2F (*I*^2^ = 44%, *p* = 0.114). Publication bias was evaluated using Egger’s regression test. All data did not show an obvious risk of publication bias (Figure 2A: *p* = 0.11, 2B: *p* = 0.10, 2C: *p* = 0.84, 2D: *p* = 0.66, 2E: *p* = 0.30, 2F: *p* = 0.76, 2G: *p* = 0.56, 2H: *p* = 0.57). Data that were included in this meta-analysis are shown in Supplementary Table S1.

## 3. Discussion

Recent studies have highlighted the potential of blood-derived parameters as biomarkers for timely patient triage, therapeutics, and stroke mechanisms [30,31,32]. Easily accessible fluid biomarkers can provide an objective evaluation of the real-time panorama, supporting stroke diagnosis or predicting the patients’ outcome and ultimately guiding clinical decisions [20,32,33,34,35]. Cognitive impairment tends to progressively worsen following stroke, with 20–30% of patients developing dementia [9,36]. Hence, international guidelines recommend cognitive assessment as a routine neurological examination for all stroke survivors [37]. Cognitive function refers to mental processes that are crucial for conducting activities of daily living. Such mental processes include attention, short-term and long-term memory, reasoning, coordination of movement, and planning of tasks [38]. The prevalence of brain disorders affecting cognition, such as stroke and dementia, increases steadily in a linear fashion with age [39]. Therefore, we evaluated the levels of eight proteins in the peripheral blood as biomarkers for stroke, especially related to brain damage and cognitive impairments.

First, we investigated whether the levels of four plasma lipids could be potential biomarkers for PSCI individually. As shown in Figure 2, the plasma levels of both TC and LDL-C were higher in the PSCI group than in the non-PSCI group. However, there was no difference in the HDL-C and TG levels between them. In fact, disorders of lipid homeostasis are common risk factors for cardiovascular diseases, which are linked to Alzheimer’s disease (AD) (Dement, 2016). Abnormal levels of lipids or lipoproteins in the blood, which include high levels of LDL-C and low levels of HDL-C, TC, and TG, were related to the cause of the disease outbreak [40,41]. Cholesterol, an important constituent of mammalian cell membranes, modulates membrane fluidity and permeability. It is also the precursor of all steroid hormones and bile acids and plays a key role in membrane trafficking, transmembrane signaling processes, and cell proliferation [42,43,44,45,46]. Cholesterol is typically transported as lipoproteins, which consist of TG and cholesterol in the center surrounded by a phospholipid shell with apolipoprotein embedded in them [47,48]. The relationship between lipids and cognition is controversial. Peripheral cholesterol levels have been evaluated in some studies [13,49,50,51,52]; Kumral et al., (2015). Most studies did not find any association between cholesterol levels and PSCI [13,50,51,52]; however, two studies found a significant association. One study showed that higher levels of LDL-C and lower levels of HDL-C were independently associated with PSCI [53]. The TG levels were studied in only four studies [49,51,52,53]. One study found an association between the baseline TG levels and PSCI [49,54]. Nevertheless, in the central nervous system (CNS), altered levels of cholesterol appear to be involved in several neurodegenerative diseases, such as AD, Niemann–Pick C disease, and major depressive disorder [55,56,57]. In addition, cholesterol-driven inflammation seems to affect neuroplasticity, altering membrane fluidity and permeability, vesicular trafficking and monoamine release, and neuroendocrine function [57,58,59,60,61]. The possibility that the differentiation and function of the CNS were partly influenced by the level of LDL-C or HDL-C circulating in the plasma received further support from recent developments in our understanding of the molecular events involved in transmembrane cholesterol movement [62]. Therefore, we insist that the TC and LDL-C levels could be potential biomarkers for PSCI, and understanding the prognostic impact of these levels in relation to PSCI may be clinically relevant. Although our results on the HDL-C and TG levels were not significant, it is necessary to consider them in future research.

Second, the levels of Hcy were higher in the PSCI group than in the non-PSCI group, as shown in Figure 2. Hcy is produced in all cells and is involved in the metabolism of cysteine and methionine [63]. In previous cross-sectional and prospective studies, elevated plasma Hcy levels have been associated with more than 100 diseases, syndromes, or outcomes [64]. Hcy metabolism is largely dependent on B vitamins, including folate, vitamin B12, and vitamin B6. Substantial lowering of the Hcy level can be achieved through B vitamin supplementation, suggesting a safe and inexpensive intervention strategy for reducing age-related cognitive decline and the risk of AD and overall dementia [65]. Whether Hcy contributes to cerebrovascular changes, cognitive function, or both remains controversial; nevertheless, it appears that elevated Hcy levels are a risk factor for dementia in older adults, regardless of the mechanism. The relationship between the Hcy levels and cognition is controversial. Although there were negative results that suggest that the Hcy levels were not associated with cognitive decline after stroke [49,66], it is a known fact that a high Hcy level is an independent risk factor for cerebrovascular events [67,68] and cognitive impairment [69,70,71,72]. In addition, a recent study showed that patients with higher Hcy levels had greater cortical and hippocampal atrophy than those with lower levels [73]. Therefore, the Hcy level could be a potential biomarker for PSCI, and understanding its prognostic impact in relation to PSCI may be clinically relevant.

Third, the levels of CRP were higher in the PSCI group than in the non-PSCI group (Figure 2). CRP is a plasma protein synthesized by the liver and is often used as a nonspecific biomarker of inflammation. Elevated CRP levels are associated with an increased risk of cerebrovascular diseases and dementia [24]. Upregulation of CRP is considered a marker of systemic inflammation in autoimmune conditions, infection, and obesity [74]. Thus, CRP plays dual roles as a marker of inflammation and a driver of the induction of inflammation [75]. There is evidence that it infiltrates the brain following BBB disruption by hemorrhagic or ischemic stroke [76,77,78]. CRP in ischemic tissues drives the progression of AD following ischemic stroke [75,79]. The relationship between the CRP level and cognition is controversial. Inconsistent results on the link between specific cognitive functions and proinflammatory markers have been reported. For example, the CRP levels have been demonstrated to be negatively related to performance in tests of episodic memory and to be predictive of poorer memory performance in some studies [80,81], but not in all [82,83]. Nevertheless, increased CRP levels may cause cognitive impairment. A previous study has found an independent association between the CRP levels and PSCI [84]. In addition, the CRP levels were negatively associated with a composite score of executive function and processing speed [85]. Therefore, the CRP level could be a potential biomarker for PSCI, and understanding its prognostic impact in relation to PSCI may be clinically relevant.

Fourth, there was no difference in the HbA1c and uric acid levels between the PSCI and non-PSCI groups. The relationship between the HbA1c levels and cognition is controversial. The HbA1c levels were evaluated in some studies. One study showed no significant association with PSCI [13]; another study showed an independent association between higher HbA1c levels and PSCI [86]. Further, there has been an accumulation of studies investigating the link between the uric acid levels and neurodegenerative diseases, mainly including dementia, Parkinson’s disease, amyotrophic lateral sclerosis, and multiple system atrophy [87]. A study of cognitively healthy adults found that elevated baseline serum uric acid levels were associated with decreased attention and visuospatial abilities in men [88]. Although our results on the HbA1c and uric acid levels were not significant, it is necessary that these levels be considered in future research.

In addition to the eight proteins selected in our study, we demonstrated that many other proteins could be considered as possible biomarkers; however, we could not meta-analyze these proteins because of insufficient data, as shown in Table 2. Many potential biomarkers for PSCI have been suggested. (1) The levels of the blood and vascular-function-related proteins, such as D-dimer, endostatin, fibrinogen, and VCAM-1, were higher in patients with PSCI than in those without; (2) the levels of the inflammatory and immune-function-related proteins, such as esRAGE, indoleamine 2,3-dioxygenase, IL-10, IL-1β, IL-6, kynurenine, kynurenine/tryptophan ratio, MMP-9, phospholipase A2, quinolinic acid, quinolinic acid/kynurenic acid ratio, RF, sRAGE, SSAO, TIMP-1, TMAO, TNF-α, and BChE, significantly differed between patients with PSCI and those without; (3) the levels of the metabolic-function-related proteins, such as FBG, non-HDL-C, and betaine, significantly differed between them; (4) the levels of the neuronal-function-related proteins, such as BACE1, neprilysin, NfL, BDNF, Aβ42, and Aβ42/Aβ40, also significantly differed between them; (5) the levels of the kidney-function-related proteins, such as cystatin C and epidermal growth factor receptor, also significantly differed between them; (6) the levels of the oxidative-stress-related proteins, such as 8-OHdG, D-amino acid oxidase, and malondialdehyde, were higher in patients with PSCI than in patients with stroke only; (7) the levels of the hormones, such as NT-proBNP, cortisol, 25(OH)D, FT4, and T3, significantly differed between patients with PSCI and those without; and (8) the levels of the other compounds, such as choline, formaldehyde, and NO^−2^, were lower in patients with PSCI than in those without. Nevertheless, further research on these candidate biomarkers is needed to validate, identify, and introduce useful biomarkers for stroke recurrence or diagnosis in a scalable manner in medical practice [17].

This study had certain limitations. Although potential blood biomarkers for PSCI were collected in various study groups, the meta-analysis was performed using available data between the PSCI and the non-PSCI group. Furthermore, this study did not consider the characteristics of the patient groups, such as the stage and duration of stroke. It has been addressed that the meta-analysis itself has poor quality of included studies, heterogeneity, and publication bias [89]. However, our study is obviously meaningful in selecting new potential biomarkers for PSCI because meta-analysis can be conceived as a systematic study of all studies that have been conducted to answer a specific question or hypothesis [90]. Our study also included patients after stroke onset, regardless of comparison with other neurodegenerative diseases. Therefore, further research is needed to compare patients with stroke with patients with mild cognitive impairment in the early stage of cognitive decline or with patients with other neurodegenerative diseases.

Nevertheless, our study demonstrated that the levels of some proteins, including TC, LDL-C, Hcy, and CRP, remarkably changed in patients with post-stroke cognitive impairment. Therefore, some blood-derived proteins, such as TC, LDL-C, Hcy, and CRP, could be potential biomarkers for the diagnosis, prognosis prediction, and progression evaluation of stroke, especially related to brain damage and cognitive impairments.

## 4. Materials and Methods

### 4.1. Literature Search and Selection Criteria

This systematic review was performed in accordance with the Preferred Reporting Items for Systematic Reviews and Meta-Analyses guidelines. All publications that described the association between PSCI and human blood biomarkers were searched in PubMed, Embase, the Web of Science, and the Cochrane Library from inception to 26 July 2021. In PubMed, we used the following search terms: (stroke OR stroke [Mesh] OR “cerebral infarct” OR “brain infarct” OR “cerebral hemorrhage” OR “cerebral hemorrhage” OR “cerebral ischemia” OR “cerebral ischemia” OR “cerebral hematoma” OR “cerebral hematoma” OR “brain hemorrhage” OR “brain hemorrhage”) AND (dementia OR dementia [Mesh] OR “cognitive decline” OR “cognitive impairment” OR cognition disorder OR cognition disorders [Mesh]) AND (biomarker OR biomarker [Mesh]) AND (blood OR serum OR plasma). In Embase, the Web of Science, and the Cochrane Library, the following terms were used: (stroke OR “cerebral infarct” OR “brain infarct” OR “cerebral hemorrhage” OR “cerebral hemorrhage” OR “cerebral ischemia” OR “cerebral ischemia” OR “cerebral hematoma” OR “cerebral hematoma” OR “brain hemorrhage” OR “brain hemorrhage”) AND (dementia OR “cognitive decline” OR “cognitive impairment” OR “cognition disorder”) AND (biomarker) AND (blood OR serum OR plasma). The inclusion criteria were as follows: (1) articles that included patients with cognitive impairment or dementia after stroke; and (2) articles that identified blood biomarkers for cognitive function after stroke.

### 4.2. Data Extraction and Analysis

Two authors (K.Y. Kim and K.-A. Chang) independently screened and selected relevant studies according to the inclusion criteria. Any disagreements on every step were resolved via constant discussion with all authors. The following relevant data were extracted from the 40 selected studies: article information, including the title, name of first author, year of publication, and country of patients; group information, including the types of group, sample size, sex, age, and measurement tool used for evaluating cognitive function; and biomarker information, including the type of specimen and type and level of potential biomarkers. For the meta-analysis, the standardized mean difference (SMD) in the potential biomarkers for evaluating PSCI was analyzed between patients with and without PSCI using the Comprehensive Meta-Analysis software version 3 (Biostats Inc., Englewood, NJ, USA). A fixed- or random-effects model was used after analyzing the Q statistic, while the *I*^2^ method was applied to assess heterogeneity. Funnel plots and Egger’s intercept tests were used to evaluate publication bias. The statistical significance level was set at *p*-values of < 0.05.

## Figures and Tables

**Table 1:** Summary of the 40 selected studies reporting potential biomarkers for PSCI.

Author and Year	Country	Study Groups	Sample Size (M/F)	Age (y)	Outcome Measurement Tool	Specimen	Potential Biomarkers
Bunevicius et al., 2015	Lithuania	Acute ischemic stroke	53/25	72	MMSE	Serum	NT-proBNP, IL-6, hs-CRP
		Hemorrhagic stroke					
Casas et al., 2017	Argentina	Control	20/20	70 ± 3/77 ± 1	MoCA	Plasma	BDNF, NO^−2^
		Acute ischemic stroke	20/20	72 ± 4/83 ± 2			
Chei et al., 2014	Japan	Control	88/104	62.2 ± 4.4	The dementia status was classified into six ranks.	Serum	hs-CRP
		Dementia with a history of stroke	44/52	62.4 ± 4.3			
		Control	98/260	62.8 ± 5.6			
		Dementia without a history of stroke	49/130	63.1 ± 5.6			
Chen et al., 2019a	Taiwan	Post-stroke without dementia	56/31	62.98 ± 9.23	CDR	Plasma	BChE
		Post-stroke dementia	18/12	73.20 ± 8.68			
Chen et al., 2019b	Taiwan	Post-stroke without dementia	41/12	61.7 ± 8.95	MMSE	Plasma	D-amino acid oxidase
		Post-stroke dementia	11/9	69.35 ± 7.24			
Choi et al., 2020	USA	Acute ischemic stroke alone	27/8	64.5 ± 14.1		Serum	IL-6, CRP, complement component 3, S100B
		Acute ischemic stroke and underlying dementia	5/3	85.8 ± 9.6			
Cogo et al., 2021	France	Post-stroke cognitive decline	6/4	64.7 ± 13.3	MMSE	Serum	Quinolinic acid, quinolinic acid/kynurenic acid ratio, tryptophan, kynurenine, kynurenic acid, kynurenine/tryptophan ratio, indoleamine 2,3-dioxygenase
		Post-stroke cognitive decline	8/5	69.4 ± 17.8			
El Hussini et al., 2020	USA	Small-vessel-type stroke	9/13	56.5 (49.5–62.0)	A standardized battery of neuropsychological tests	Plasma	VCAM-1, IFN-γ, IL-1 RA, IL-6, IL-8, IL-10, thrombin-antithrombin
Feng et al., 2020	China	Stroke rhGH group	18/8	61.3 ± 10	MoCA	Plasma	TC, LDL-C, HDL-C, TG, FBG, HbA1c, IGF-1, VEGF
		Stroke placebo group	17/9	60.8 ± 11.3			
Ge et al., 2020	China	Acute ischemic stroke	414/184	59.9 ± 10.5	MMSE/MoCA	Serum	TIMP-1, MMP-9
Gold et al., 2011	Canada	Ischemic stroke	22/19	72.3 ± 12.2	MMSE	Plasma	Tryptophan, L-kynurenine, L-kynurenine/tryptophan
Hou et al., 2019	China	Total stroke	140/121	66.4 ± 9.3	MoCA	Serum	TC, TG, LDL-C, HDL-C, hs-CRP, Hcy, retinoic acid
		Stroke without PSCI	65/55	67.7 ± 9.3			
		Stroke with PSCI	75/66	67.7 ± 9.3			
Kliper et al., 2013	Israel	First-ever mild to moderate stroke			MoCA	Serum	CRP
Krzystanek et al., 2007	Poland	Stroke	15/17	74.13 ± 7.43	MMSE	Platelet	Phospholipase A2
		Vascular dementia	13/19	75.25 ± 9.22			
		Alzheimer’s disease	10/27	73 ± 6.45			
Kulesh et al., 2018	Russian Federation	Normal cognition	8/7	59.5 ± 10.0	MMSE/MoCA	Serum	IL-1β, IL-6, IL-10, TNFα
		Dysexecutive cognitive impairment	13/8	66.4 ± 8.8			
		Mixed cognitive impairment	16/5	67.8 ± 8.2			
Liu et al., 2018	China	Acute ischemic stroke	71/37		MMSE	Plasma	Uric acid, creatinine, urea N, glucose
		Better outcome (mRS score of ≤ 2)	32/19	63.9 ± 14.9			
		Poor outcome (mRS score of >2)	39/18	66.1 ± 16.2			
Liu et al., 2017	China	Non-PSCI	65/27	60 (52.3–65.8)	MMSE	Serum	Malondialdehyde, 8-OHdG
		PSCI	56/45	66 (56–72)			
Lu et al., 2016	China	Acute ischemic stroke	192/61		MMSE/MoCA		Non-HDL-C, TC, HDL-C, LDL-C, FBG, TG, Hcy, hs-CRP, HbA1c
		Normal non-HDL-C		63.1 ± 11.9			
		High non-HDL-C		62.2 ± 10.8			
Mao et al., 2020	China	Non-PSCI	79/37	65 (60–74)	MoCA	Serum	Aβ42, T3, T4, FT3, FT4, TSH, TC, TG, HDL-C, LDL-C, hs-CRP, Hcy
		PSCI	38/34	73 (66–80)			
Marklund et al., 2004	Sweden	Acute ischemic stroke	56/32	71 ± 11	MMSE	Serum	Cortisol, DS, cortisol/DS ratio
Pedersen et al., 2018	Sweden	Acute ischemic stroke	169/99	18–69	BNIS	Plasma/serum	Von Willebrand factor, tissue plasminogen activator, fibrinogen, hs-CRP
		Stroke for <50 years	32/35				
Qian et al., 2012	China	Stroke	44/20	62.1 ± 1.6	MMSE/MoCA	Serum	sRAGE, BACE, neprilysin
		Vascular cognitive impairment with no dementia	19/18	65.5 ± 1.7			
		Vascular dementia	18/18	73.8 ± 2.1			
		Mixed dementia	6/9	74.6 ± 2.2			
Qian et al., 2020	China	Endostatin concentration group	431/182	60.0 ± 10.5	MoCA	Plasma	Endostatin
Ran et al., 2020	China	Stroke	41/74	57.72 ± 6.11	MoCA	Serum	Uric acid, hs-CRP, fibrinogen, TG, cholesterol
		PSCI	43/39	59.99 ± 7.46			
Stokowska et al., 2021	Sweden	Intervention group	64/51		Letter number sequence test	Plasma	NfL
Sun et al., 2020	China	Non-PSCI	60/26	64.66 ± 11.57	MoCA	Serum	Uric acid, folic acid, VB12, Hcy, TG, cholesterol, HDL-C, LDL-C
		PSCI	110/78	71.3 ± 10.88			
Tang et al., 2017	Taiwan	Stroke without vascular dementia	90/46	71.2 ± 6.9	CDR/MMSE/MoCA	Plasma	sRAGE, esRAGE
		Stroke with vascular dementia	21/15	75.4 ± 8.8			
Tong et al., 2017	China	Stroke	21/21	75.55 ± 2.39	MMSE	Plasma	Semicarbazide-sensitive amino oxidase, formaldehyde
		Post-stroke dementia	21/21	76.14 ± 3.73			
Wang et al., 2021	China	Stable	148/107	64.86 ± 9.37	MMSE/MoCA	Serum	NfL
		Progression	26/23	65.18 ± 8.61			
Wang et al., 2020	China	Control	14/16	66.1 ± 5.9	MMSE/MoCA	Plasma/serum	Aβ40, Aβ42, Aβ42/Aβ40, CRP, TNF-α, IL-6
		Observation	17/13	67.2 ± 7.1			
Wang et al., 2021	China	Non-PSCI	355/200	62 ± 13	MoCA	Plasma	pNfL, HbA1c, hs-CRP, Hcy
		PSCI	538/491	66 ± 18.5			
Weng et al., 2020	China	Non-PSCI	130/67	64	MoCA	Blood	CRP, TB, DBIL, IBIL, TC, Ca, uric acid, HbA1c, D-dimer
		PSCI	102/74	72			
Yalbuzdag et al., 2015	Turkey	Ischemic	53/43	63.78 ± 12.3	MMSE	Plasma	25(OH)D
		Hemorrhagic	11/13	61.8 ± 10.0			
Yan et al., 2015	China	Non-vascular dementia	56/48		MMSE/MoCA	Serum	Hcy, hs-CRP, LDL-C
		Vascular dementia					
Zeng et al., 2019	China	Cognitive impairment no dementia	61/20	71.40 ± 11.32	MoCA	Serum	Cystatin C, HbA1c, creatinine, uric acid, TC, TG, HDL-C, LDL-C
		Vascular cognitive impairment	45/26	76.28 ± 15.16			
Zhong et al., 2018	China	MMP concentration group	558		MMSE/MoCA	Serum	MMP-9
Zhong et al., 2021	China	Choline/betaine/TMAO	433/184	60 ± 10.5	MMSE/MoCA	Plasma	Choline, betaine, TMAO
Zhu et al., 2020	China	Non-PSCI	89/81	65 ± 10.8	MMSE	Plasma	TMAO, TC, TG, LDL-C, HDL-C, hs-CRP, FBG, Hcy
		PSCI	50/36	71.1 ± 10.4			
Zhu et al., 2019	China	RF concentration group	582		MMSE/MoCA	Serum	RF
Zhu et al., 2019	China	MMSE/MoCA group	448/190	60.7 ± 10.3	MMSE/MoCA	Serum	aPS, GPS, aCL, GPL, β2-GPI, RF, NT-proBNP, Lp-PLA2 mass, MMP-9, tHcy, eGFR, uric acid, HGF

25(OH)D: 25-hydroxyvitamin D3, 8-OHdG: 8-hydroxydeoxyquanosine, aCL GPL: anticardiolipin antibodies, IgG anticar-diolipin antibodies units, aPS GPS: anti-phosphatidylserine antibodies, IgG antiphosphatidylserine antibodies units, Aβ40: amyloid β 40, Aβ42: amyloid β 42, BACE: β-secretase enzyme, BChE: butyrylcholinesterase, BDNF: brain-derived neurotrophic factor, CDR: clinical dementia rating, CRP: C-reactive protein, DBIL: direct bilirubin, DS: dehydroepiandrosterone sulphate, eGFR: estimated glomerular filtration rate, esRAGE: endogenous secretory RAGE, FBG: fasting blood glucose, FT3: free triiodothyroinine, FT4: free thyroxin, HbA1c: glycated hemoglobin, Hcy: homocysteine, HDL-C: high-density lipoprotein cholesterol, HGF: hepatocyte growth factor, hs-CRP: high-sensitivity C-reactive protein, IBIL: indirect bilirubin, IFN-γ: interferon-gamma, IGF-1: insulin-like growth factor-1, IL-1 RA: interleukin-1 receptor antagonist, IL-10: interleukin-10, IL-1β: interleukin-1 beta, IL-6: interleukin-6, IL-8: interleukin-8, LDL-C: low-density lipoprotein cholesterol, Lp-PLA2: lipoprotein-associated phospholipase A2, MMP-9: matrix metalloproteinase-9, MMSE: Mini-Mental State Examination, MoCA: Montreal Cognitive Assessment, NfL: neurofilament light, NT-proBNP: N-terminal pro b-type natriuretic peptide, PSCI: post-stroke cognitive impairment, RF: rheumatoid factor, sRAGE: soluble receptor for advanced glycation end products, T3: triiodothyronine, T4: thyroxin, TB: total bilirubin, TC: total cholesterol, TG: triglyceride, tHcy: total homocysteine, TIMP-1: tissue inhibitor metalloproteinase-1, TMAO: trimethylamine N-oxide, TNFα: tumor necrosis factor-alpha, TSH: thyrotropin, VB12: vitamin B12, VCAM-1: vascular cell adhesion molecule 1, VEGF: vascular endothelial growth factor, β2-GPI: beta(2)-glycoprotein 1-dependent anticardiolipin antibodies.

**Table 2:** Changes in the potential blood biomarkers for PSCI.

Category	Level	Potential Biomarkers
Blood and vascular functions	Increase	D-dimer, Hcy, endostatin, fibrinogen, VCAM-1
	No change	Direct bilirubin, fibrinogen, Hcy, indirect bilirubin, total bilirubin, tissue plasminogen activator, vitamin B12, VEGF, von Willebrand factor, thrombin-antithrombin
Inflammatory and immune functions	Increase	esRAGE, hs-CRP (CRP), indoleamine 2,3-dioxygenase, IL-10, IL-1β, IL-6, kynurenine, MMP-9, phospholipase A2, quinolinic acid, RF, sRAGE, semicarbazide-sensitive amino oxidase, TIMP-1, TMAO, TNF-α, kynurenine/tryptophan ratio, quinolinic acid/kynurenic acid ratio
	Decrease	BChE, hs-CRP (CRP), sRAGE
	No change	aCL GPL, aPS GPS, β2-GPI, complement component 3, hs-CPR (CRP), kynurenic acid, Lp-PLA2 mass, tryptophan, IFN-γ, IL-1 RA, IL-6, IL-8, IL-10
Metabolic function	Increase	FBG, HbA1c, HDL-C, LDL-C, non-HDL-C, TC, TG
	Decrease	Betaine, TC levels
	No change	FBG, glucose, HbA1c, HDL-C, HGF, LDL-C, TC, TG, IGF-1
Neuronal function	Increase	BACE1, neprilysin, NfL
	Decrease	BDNF, Aβ42, Aβ42/Aβ40, NfL
	No change	S100B, Aβ42, Aβ40, AChE, neprilysin
Kidney function	Increase	Cystatin C, uric acid
	Decrease	eGFR, uric acid
	No change	Creatinine, uric acid, urea N
Oxidative stress	Increase	8-OHdG, D-amino acid oxidase, malondialdehyde
Hormone	Increase	NT-proBNP, cortisol
	Decrease	25(OH)D, FT4, T3
	No change	Cortisol/DS ratio, DS, FT3, NT-proBNP, T4, TSH
Others	Decrease	Choline, formaldehyde, NO^−2^
	No change	Ca, folic acid, TMAO, retinoic acid

25(OH)D: 25-hydroxyvitamin D3, 8-OHdG: 8-hydroxydeoxyquanosine, aCL GPL: anticardiolipin antibodies, IgG anticar-diolipin antibodies units, aPS GPS: anti-phosphatidylserine antibodies, IgG antiphosphatidylserine antibodies units, Aβ40: amyloid β 40, Aβ42: amyloid β 42, β2-GPI: beta(2)-glycoprotein 1-dependent anticardiolipin antibodies, BACE: β-secretase enzyme, BChE: butyrylcholinesterase, BDNF: brain-derived neurotrophic factor, CDR: clinical dementia rating, CRP: C-reactive protein, DS: dehydroepiandrosterone sulphate, eGFR: estimated glomerular filtration rate, esRAGE: endogenous secretory RAGE, FBG: fasting blood glucose, FT3: free triiodothyroinine, FT4: free thyroxin, HbA1c: glycated hemoglobin, Hcy: homocysteine, HDL-C: high-density lipoprotein cholesterol, HGF: hepatocyte growth factor, hs-CRP: high-sensitivity C-reactive protein, IFN-γ: interferon-gamma, IGF-1: insulin-like growth factor-1, IL-1 RA: interleukin-1 receptor antagonist, IL-10: interleukin-10, IL-1β: interleukin-1 beta, IL-6: interleukin-6, IL-8: interleukin-8, LDL-C: low-density lipoprotein cholesterol, Lp-PLA2: lipoprotein-associated phospholipase A2, MMP-9: matrix metalloproteinase-9, MMSE: Mini-Mental State Examination, MoCA: Montreal Cognitive Assessment, NfL: neurofilament light, NT-proBNP: N-terminal pro b-type natriuretic peptide, PSCI: post-stroke cognitive impairment, RF: rheumatoid factor, sRAGE: soluble receptor for advanced glycation end products, T3: triiodothyronine, T4: thyroxin, TC: total cholesterol, TG: triglyceride, tHcy: total homocysteine, TIMP-1: tissue inhibitor metalloproteinase-1, TMAO: trimethylamine N-oxide, TNFα: tumor necrosis factor-alpha, TSH: thyrotropin, VB12: vitamin B12, VCAM-1: vascular cell adhesion molecule 1, VEGF: vascular endothelial growth factor.

## Data Availability

The data that support the findings of this study are available from the corresponding authors upon reasonable request.

## Supplementary Materials

The following are available online at https://www.mdpi.com/article/10.3390/ijms23020602/s1.
