# Supplementary material for: Potential Biomarkers for Post-Stroke Cognitive Impairment: A Systematic Review and Meta-Analysis
Source: Int J Mol Sci. 2022 Jan 6;23(2):602. doi: 10.3390/ijms23020602 (PMC8775398; doi:10.3390/ijms23020602)
Supplement: Supplementary file 1 [file ijms-23-00602-s001.zip › ijms-1481616-Supplementary table1.pdf]

Supplementary Table S1. Summary of the included studies for meta-analysis

Biomarker	Study	group characteristics				Std diff in means	95% CI	p	Model
		PSCI group		Non-PSCI group					
		n	M±SD or mean	n	M±SD or mean				
Hcy	Hou et al., 2019	141	15.4±5.3	120	14.1±4.9	0.254	0.010~0.498	0.042	Random effect
	Mao et al., 2020	72	15.8±8.2	116	15±8.6	0.095	-0.200~0.389	0.528	
	Sun et al., 2020	188	13.16±7.66	86	11.14±4.3	0.297	0.041~0.554	0.023	
	Wang et al., 2021	1029	15.18	6650	14.64	0.110	0.045~0.176	0.001	
	Yan et al., 2015	91	21±4.21	13	13±5.03	1.854	1.221~2.488	0.000	
	Zhu et al., 2020	86	15.5±4.9	170	14.3±5.4	0.229	-0.031~0.489	0.084	
CRP	Choi et al., 2020	8	19.8	35	3.5	1.237	0.425~2.048	0.003	Random effect
	Hou et al., 2019	141	5.3	120	5.5	0.053	-0.190~0.297	0.668	
	Mao et al., 2020	72	4.8±4.6	116	6.2±7.1	0.223	-0.071~0.518	0.137	
	Ran et al., 2020	82	10.7±5.3	115	6.2±2.7	1.127	0.823~1.432	0.000	
	Wang et al., 2021	1029	3.51	665	3.61	0.048	-0.049~0.146	0.332	
	Weng et al., 2020	176	2.89	197	2.2	0.287	0.082~0.491	0.006	
	Yan et al., 2015	91	6.35±1.96	13	6.07±2.35	0.139	-0.442~0.721	0.639	
	Zhu et al., 2020	86	6.8	170	3.4	0.367	0.105~0.628	0.006	
Uric acid	Ran et al., 2020	82	291.8±98.3	115	326.4±103.7	-0.341	-0.626~-0.056	0.019	Random effect
	Sun et al., 2020	188	342.83±89.14	86	311.8±69.29	0.372	0.115~0.629	0.005	
	Weng et al., 2020	176	302.5	197	311	-0.091	-0.294~0.113	0.383	
	Zeng et al., 2019	71	341.14±61.9	81	328.8±77.06	0.175	-0.144~0.495	0.282	
HbA1c	Wang et al., 2021	1029		665		0.006	-0.091~0.104	0.901	Fixed

	Weng et al., 2020	176	6.3	197	6.1	0.181	-0.023~0.385	0.082	
	Zeng et al., 2019	71	7.1±1.19	81	7.09±1.9	0.006	-0.312~0.325	0.969	
TC	Hou et al., 2019	141	4.1±1.1	120	3.9±1	0.190	-0.054~0.434	0.128	Fixed
	Mao et al., 2020	72	4.54±0.78	116	4.58±0.72	0.054	-0.240~0.348	0.720	
	Ran et al., 2020	82	5.32±1.02	115	5.25±0.96	0.071	-0.212~0.354	0.623	
	Sun et al., 2020	188	4.37±1.06	86	4.47±1.06	0.094	-0.161~0.350	0.469	
	Zeng et al., 2019	71	5.39±0.97	81	4.83±1.32	0.479	0.156~0.802	0.004	
	Zhu et al., 2020	86	4±1.1	170	4±1	0.000	-0.259~0.259	1.000	
TG	Hou et al., 2019	141		120		0.081	-0.163~0.324	0.515	Fixed
	Mao et al., 2020	72	1.48±0.57	116	1.31±0.41	0.356	0.060~0.652	0.018	
	Ran et al., 2020	82	1.58±1.33	115	1.68±1.41	-0.073	-0.356~0.211	0.616	
	Sun et al., 2020	188	1.57±0.97	86	1.76±0.88	-0.202	-0.457~0.054	0.122	
	Zeng et al., 2019	71	1.33±0.4	81	1.37±0.57	-0.080	-0.399~0.238	0.621	
	Zhu et al., 2020	86	1.4	170	1.3	0.044	-0.216~0.303	0.741	
HDL-C	Hou et al., 2019	141	1.1±0.2	120	1.1±0.2	0.000	-0.243~0.243	1.000	Random effect
	Mao et al., 2020	72	1.19±0.17	116	1.12±0.22	0.346	0.050~0.642	0.022	
	Sun et al., 2020	188	1.08±0.3	86	1.08±0.35	0.000	-0.255~0.255	1.000	
	Zeng et al., 2019	71	1±0.33	81	1.05±0.28	0.134	-0.155~0.483	0.313	
	Zhu et al., 2020	86	1±0.2	170	1.1±0.2	0.500	0.237~0.763	0.000	
LDL-C	Hou et al., 2019	141		120		0.035	-0.209~0.278	0.780	Random effect
	Mao et al., 2020	72	2.85±0.71	116	2.34±0.76	0.688	0.386~0.990	0.000	
	Sun et al., 2020	188	1.34±0.84	86	1.35±0.88	0.012	-0.243~0.267	0.928	
	Yan et al., 2015	91	4.27±2.78	13	3.81±2.36	0.168	-0.413~0.750	0.571	

	Zeng et al., 2019	71	3.45±0.77	81	3.2±0.87	0.303	-0.017~0.624	0.064	
	Zhu et al., 2020	86	2.4	170	2.3	0.125	-0.134~0.385	0.344	

Hcy: homocysteine, CRP: C-reactive protein, HbA1c: glycated hemoglobin, TC: total cholesterol, TG: triglyceride, HDL-C: high-density lipoprotein cholesterol, LDL-C: low-density lipoprotein cholesterol, Std diff: standard difference, CI: confidence interval, PSCI: post-stroke cognitive impairment
